# Not Another Pleural Effusion: A Unique Case of Metastatic Endometrial Stromal Sarcoma

**DOI:** 10.7759/cureus.38341

**Published:** 2023-04-30

**Authors:** Andrea Ramirez, Fereshteh Yazdi, Rachaita Lakra, Jennifer Lee, Prangthip Charoenpong

**Affiliations:** 1 Pulmonology and Critical Care Medicine, Louisiana State University Health Shreveport, Shreveport, USA; 2 Internal Medicine, Louisiana State University Health Shreveport, Shreveport, USA; 3 Pathology, Louisiana State University Health Shreveport, Shreveport, USA

**Keywords:** oncology pathology, endometrial stromal sarcomas, endocrine oncology, pleural effusion, mesenchymal tumor, case report

## Abstract

The etiology of complicated pleural effusion can be vast. We present a unique case of an unsuspected metastatic endometrial stromal sarcoma (ESS) in an asymptomatic patient with an incidentally found complicated pleural effusion. A 69-year-old female with no pertinent past medical history was referred to pulmonology for an effusion noted on a routine chest X-ray. Her surgical history was significant for a hysterectomy. At the time of evaluation in the pulmonology clinic, the patient was asymptomatic with stable vital signs. Computed tomography of her chest showed a complex pleural effusion which was drained by cardiothoracic surgery. Fluid analysis results were positive for estrogen and progesterone receptor-positive mesenchymal tumor. Follow-up imaging was negative for any other metastasis. Appropriate management and drainage of this asymptomatic pleural effusion resulted in the diagnosis of a rare malignancy. Given the good clinical prognosis of mesenchymal tumors, the patient was appropriately treated and doing well.

We present the case of a patient who was found to have a rare malignancy rather than a benign chronic pleural effusion, as previously suspected. This neoplasm represented a metastatic ESS, especially in this patient’s setting of a hysterectomy.

## Introduction

The etiology of complicated pleural effusion can be vast. Malignant pleural effusions are the second most common cause of exudative effusions and are indicative of disseminated malignancy and poor clinical outcomes [1]. As a result, pleural fluid analysis and molecular biomarkers are especially helpful in accurate diagnosis and timely treatment of underlying etiology. We present a unique case of an unsuspected metastatic endometrial stromal sarcoma (ESS) in an asymptomatic patient with an incidentally found complicated pleural effusion [2].

This case was previously presented as an oral presentation during the annual Chest Conference in Nashville, Tennessee on October 19, 2022.

## Case presentation

**Figure 1:**
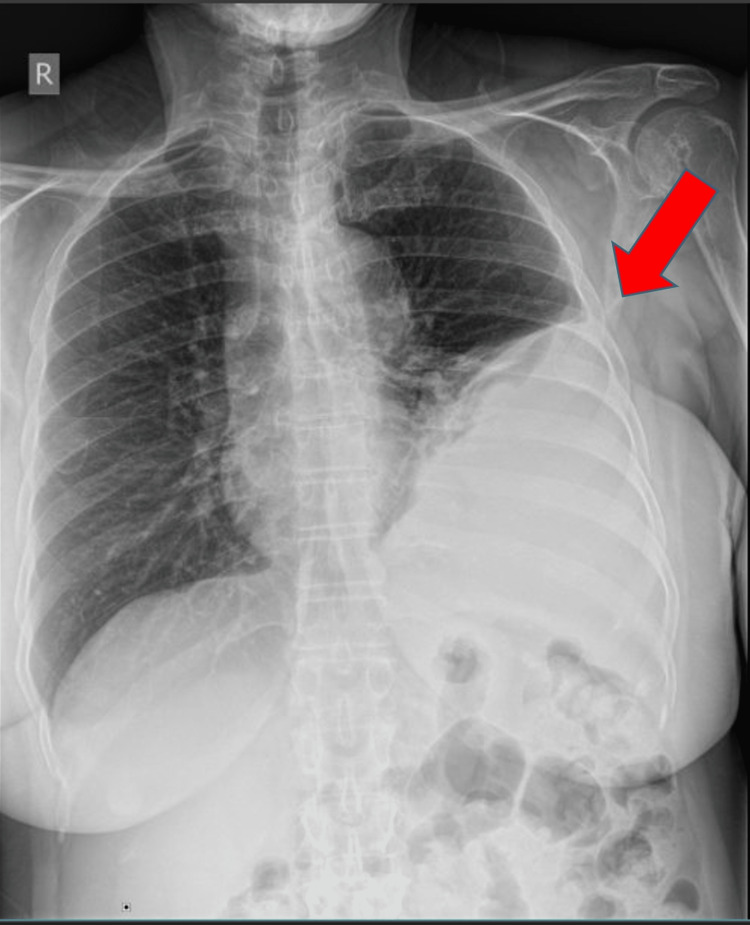
Chest X-ray showing an incidental pleural disease.

A 69-year-old female presented to the pulmonary clinic for evaluation of an abnormal chest X-ray with an incidental pleural disease that was obtained as a health maintenance examination (Figure 1).

**Figure 2:**
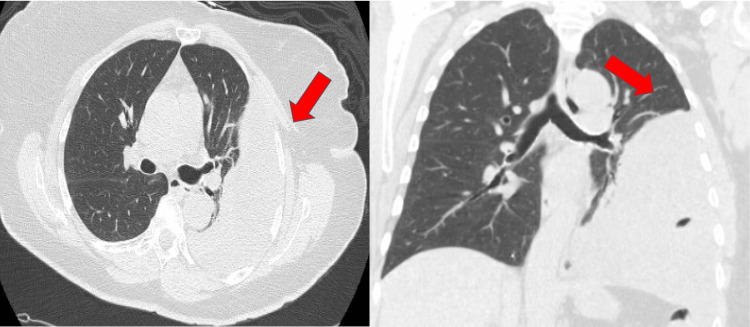
Computed tomography of the chest obtained for further evaluation of pleural disease showing complex pleural collection.

At the time of presentation, the patient was asymptomatic. Her vitals, physical examination, and laboratory findings were within normal limits. She had a non-contributory past medical history. She had a remote surgical history of hysterectomy for an unknown reason. The patient also denied any family history of endometrial and pulmonary malignancy. She denied any smoking history or exposure to secondhand smoke. A computed tomography (CT) of the chest was obtained and demonstrated a complex left pleural effusion (Figure 2).

Point-of-care ultrasound was consistent with an organized effusion, and thoracentesis was performed. Only 30 mL of fluid was obtained with fluid analysis resulting in an exudate with lymphocyte predominate. Because the effusion was well organized, cardiothoracic surgery was consulted for diagnosis and decortication.

**Figure 3:**
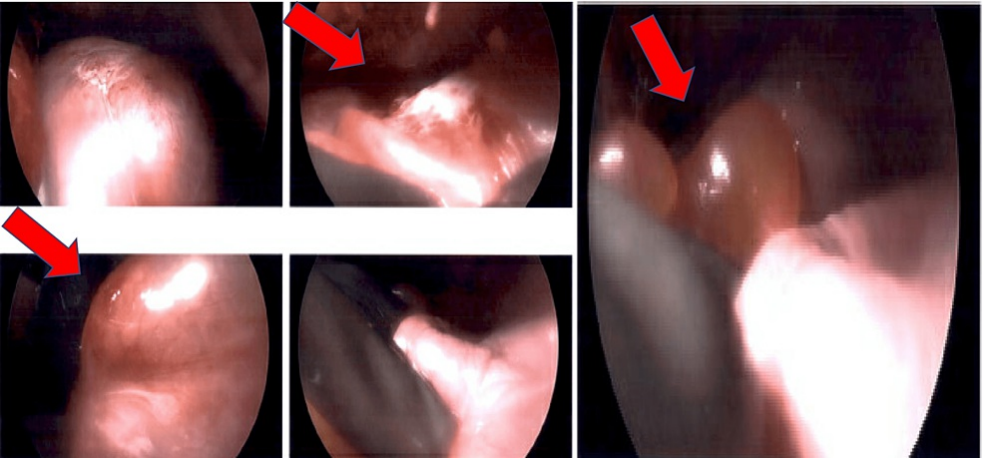
A large complex mass surrounded by mucinous cysts seen intraoperatively and occupying the entire left lower lobe of the lung. The patient subsequently underwent a lobectomy.

**Figure 4:**
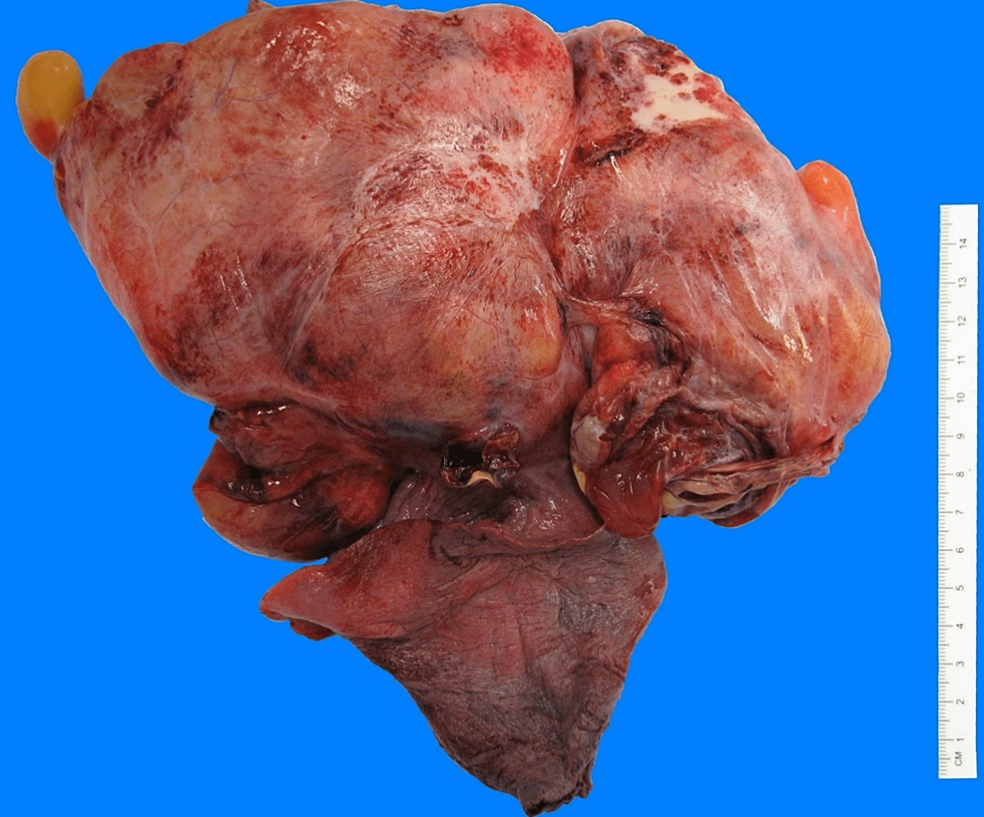
Gross pathology image of the mass after resection. The external pleural surface contained multiple large thin-walled bullae and thick fibrotic bands with prominent vasculature. The pleural surface shows areas of gray-white thickening with punctate hemorrhage.

**Figure 5:**
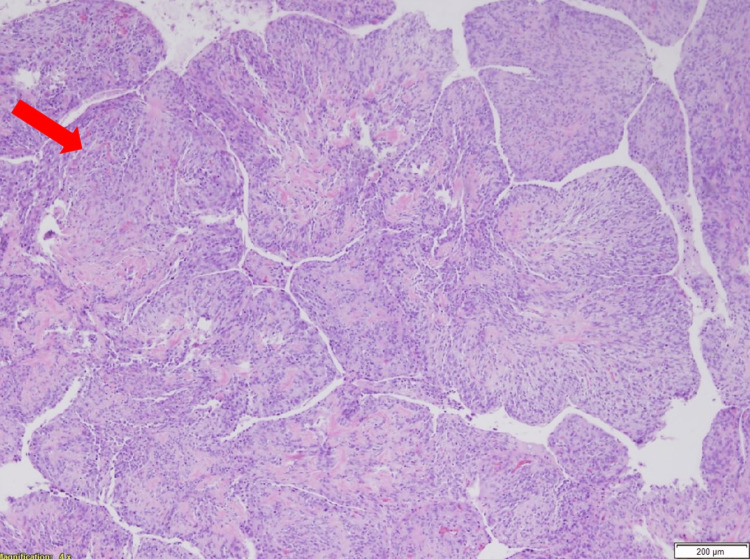
Histopathology of the cystic mass with evidence of heterogenous elements and striking phyllodes-like growth pattern in which individual lobules are surfaced by normal respiratory epithelium.

**Figure 6:**
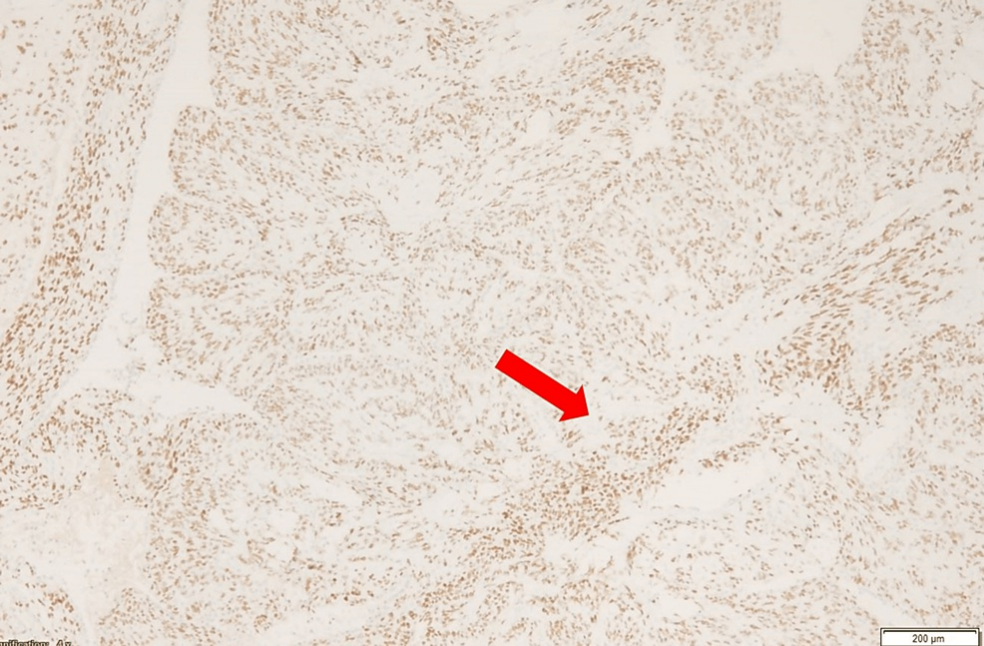
Immunostaining showing evidence of estrogen-positive receptors.

During surgery, a large complex mass surrounded by mucinous cysts was seen occupying the entire left lower lobe of the lung (Figures 3, 4). The patient subsequently underwent a lobectomy. Initial pathology results posed a diagnostic challenge and were therefore submitted to the Mayo Clinic for further analysis. Mayo Clinic identified the tumor as an estrogen and progesterone receptor-positive mesenchymal tumor with heterologous elements (Figures 5, 6).

**Figure 7:**
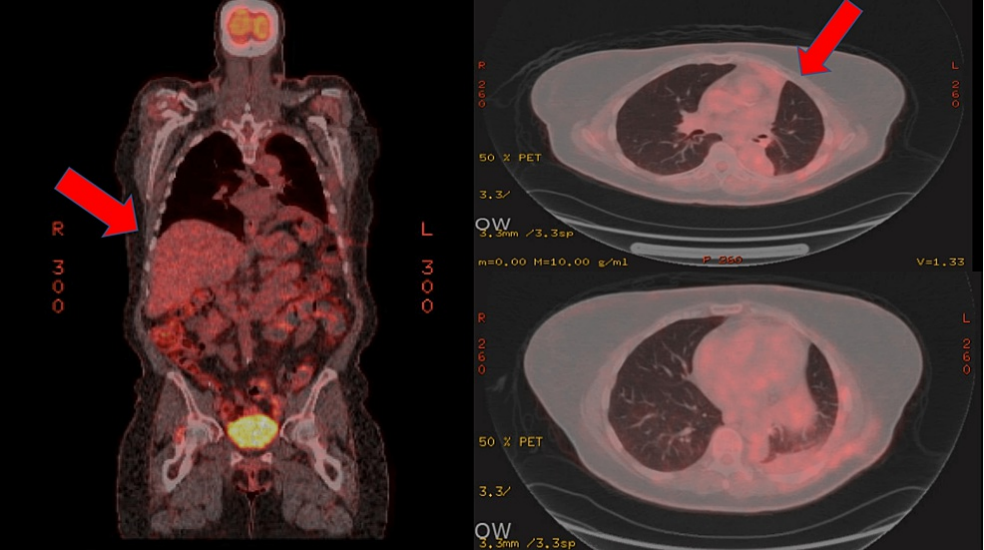
Positron emission tomography scan with no evidence of active malignancy or suspicious pulmonary nodules.

Genetic studies were negative for fusion genes. Cytology of the pleural fluid was also positive for atypical cells. On detailed analysis, she was found to have metastatic ESS. A postoperative positron emission tomography scan showed no definite evidence of either local or metastatic disease (Figure 7). She was subsequently started on hormonal therapy with excellent response to therapy. She did not undergo any chemo or radiation therapy and continues to do well during her follow-up appointments.

## Discussion

We presented the case of a 69-year-old patient with a history of hysterectomy who was found to have a rare malignancy rather than a benign chronic pleural effusion, as previously suspected. This metastatic ESS is especially intriguing in the setting of the patient’s history of hysterectomy. ESS are very rare malignant tumors that account for around 0.2% of all uterine malignancies [3]. The annual incidence of ESS is 1-2 per million women. The diagnosis is not based on imaging and requires histopathological confirmation, which can further complicate or delay diagnosis [4]. Due to the rarity of the tumors, little clinical information has been collected to expand on therapeutic and management approaches. Compared to other uterine malignancies, ESS affects younger women, and the mean age is 42 to 58 years [5]. They account for less than 10% of uterine mesenchymal neoplasms. ESS is an indolent tumor with local recurrences and distant metastasis that can occur even 20 years after the initial diagnosis, as seen in our patient. Once diagnosed, they can be treated with hormonal therapy and local radiation and tend to have a good prognosis, but definite prognostication of these tumors is hindered by the lack of large-scale clinical data [4].

## Conclusions

Due to the rare occurrence of metastatic ESS, clinical information regarding diagnosis, different presentations, and management is scarce. This unique case of metastatic ESS in an elderly asymptomatic patient highlights the importance of diagnostic studies for complicated pleural effusion. In case of complex, well-organized effusions or when there is a high clinical suspicion for malignancy, surgical interventions should be pursued in a timely manner as they can provide both diagnostic and therapeutic opportunities to improve clinical outcomes.
